# The Association between Children’s Behavior and Parenting of Caregivers: A Longitudinal Study in Japan

**DOI:** 10.3389/fpubh.2016.00017

**Published:** 2016-02-15

**Authors:** Kota Suzuki, Yosuke Kita, Makiko Kaga, Kenji Takehara, Chizuru Misago, Masumi Inagaki

**Affiliations:** ^1^Department of Developmental Disorders, National Center of Neurology and Psychiatry (NCNP), National Institute of Mental Health, Tokyo, Japan; ^2^Tokyo Metropolitan Medical Center EAST for Children/Adults with Developmental Disabilities, Tokyo, Japan; ^3^Department of Health Policy, National Center for Child Health and Development, Tokyo, Japan; ^4^Department of International and Cultural Studies, Tsuda College, Tokyo, Japan

**Keywords:** parenting, child development, developmental disorders, structural equation modeling, family psychology

## Abstract

The purpose of this study was to elucidate the association between children’s behavior (i.e., prosocial and problematic behavior) and the parenting style (i.e., laxness and overreactivity) of their caregivers by using longitudinal data in the Japanese population. These data were collected when the children were 7.5 and 9 years. We proposed three hypotheses: children’s behavior at 7.5 years will predict their behavior at 9 years; children’s behavior at 7.5 years will predict the parenting of their caregivers; and the parenting style of caregivers will affect their children’s behavior at 9 years. We evaluated children’s behavior and parenting behavior using a strength and difficulties questionnaire and a parenting scale. The hypotheses were tested using structural equation modeling (SEM). The results of the SEM showed that children’s behavior at 7.5 years predicted their behavior at 9 years. Children’s problematic behavior at 7.5 years triggered overreactive parenting in their caregivers at 9 years, which increased problematic behavior and decreased prosocial behavior in the children at 9 years. These findings indicate the association between children’s behavior and the parenting style of caregivers in Japan.

## Introduction

Children interact with their environment (e.g., family and school), and these interactions affect their development (1). Children share a home with their primary caregivers or parents for a long time. Thus, child development is influenced by their relationship to the caregiver.

Previous clinical intervention studies have made reference to the importance of the interactions between children and their caregivers. Parent training programs as one of the intervention methods aimed at teaching parents how to rear their children are effective in improving the behavior of children with developmental disorders, such as attention deficit hyperactive disorder (ADHD) (2) and autism spectrum disorder (ASD) (3). A meta-analysis showed that a parenting program, Triple P, reduced the problematic behavior of children and the effect was maintained in the long term (4). Consistent with the findings of clinical intervention studies, it has been reported that the behavioral problems of children with intellectual disability reduced when their mothers provide them with an optimal level of support and assistance (5). Positive maternal attitudes toward children lead to a reduction in children’s internalizing and externalizing problem behaviors (6), a decrease in symptoms of ASD (6), and a decrease in the risk of developing ADHD (7). Chang et al. (8) showed that mothers and fathers with a harsh parenting style contributed to their children’s emotional regulation problems. Therefore, we assumed that the parenting behaviors of the caregivers can predict children’s behavior.

Moreover, previous findings have shown a reverse direction of the effects, in that caregivers are also influenced by their children’s behavior. Compared with the caregivers of children with typical development (TD), those of children with ASD tend to face a higher risk of depression (9). Parents of children with developmental disorders behaved more inappropriately with their children than did parents of children with TD (10–13). These findings suggest that severe behavioral problems in children are associated with difficulties in child-rearing, which cause inappropriate parenting behavior (14, 15).

From these findings, it was assumed that there is a bidirectional relation between parenting behavior and children’s behavior. However, this assumption was mainly based on the findings in Western countries, although the parenting style and the effect of parenting behavior on children’s behavior were different among cultures (16–18). Hence, it was necessary to confirm the association between children’s behavior and the parenting behavior in Japanese population, which may provide evidence for a clinical intervention for caregivers in Japan.

In Japanese culture, there is a close relation between caregivers and children compared with Western country (18, 19). Moreover, Shikishima et al. (20) reported that children’s characteristic on parenting behavior had differential influences for Japanese and Western mothers, with Japanese children having a stronger influence. Therefore, we hypothesized that the parenting behavior has a stronger association with children’s behavior in Japan than in other countries.

Although parenting styles are different among cultures, the parenting scale (PS) (21) is used for the assessment of clinical practice in Japan (22) and several countries (23, 24). Similar to Western countries (25, 26), the validity of the PS was confirmed in Japan (27), which showed the reliability of two-factor structure for laxness and overreactivity. Laxness refers to the tendency to behave permissively and inconsistently when parenting children (27). The inconsistent and permissive parenting style has been reported to be associated with psychological disorders (28) and violence (29) in adolescents. Overreactivity refers to an authoritarian and harsh parenting style, in other words, one that involves using threats and harsh punishment (27). A previous study showed that the authoritarian and harsh parenting style enhances the children’s problematic behavior regardless of cultural differences (16). In Japan, mental health problems in adulthood were predicted by the authoritarian parenting style in childhood (30). Moreover, overreactivity and laxness were correlated with children’s problematic behavior in the Japanese population (27). Thus, we hypothesized that both dysfunctional styles would be associated with children’s behavior in the Japanese sample.

**Figure 1:**
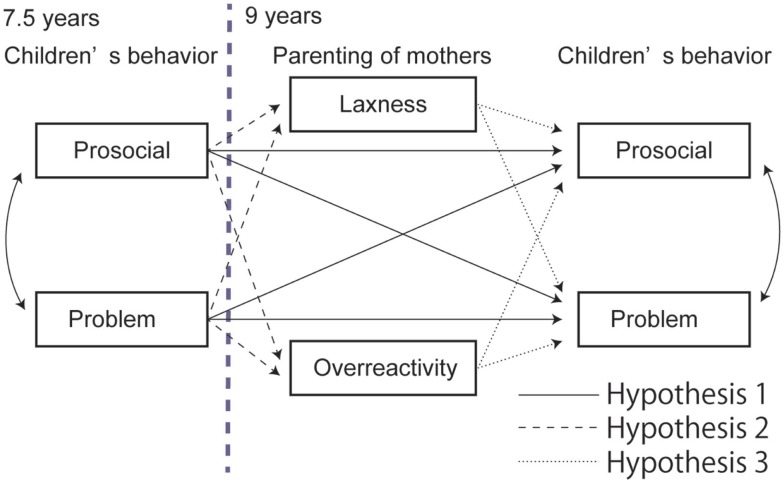
**Original proposed model of the association between children’s behavior and mothers’ parenting**.

Previous studies have shown that problematic behavior develops from early childhood to adolescence, and is associated with the parenting behavior of caregivers (31). Thus, this relationship may be seen in school-aged children. Longitudinal data were used to investigate this change over time in the Japanese population, and the children were studied at 7.5 and 9 years of age. Based on the findings mentioned earlier, we proposed three hypotheses:

1. Children’s behavior at 7.5 years will predict their behavior at 9 years (Figure 1, solid lines).
2. Children’s behavior at 7.5 years will influence laxness and overreactivity of their caregivers at 9 years (Figure 1, dashed lines).
3. Laxness and overreactivity of caregivers when their children are 9 years will influence the children’s behavior at 9 years (Figure 1, dotted lines).

Since these hypotheses were closely related to each other and needed to be comprehensively examined, we introduced structural equation modeling (SEM).

## Materials and Methods

### Participants

The current research was part of a longitudinal study, wherein the children were studied from birth; they were born in four midwifery center or a hospital in Japan from March 2002 to August 2003 (32, 33). At the follow-up study, May 2010 (i.e., the children’s ages were approximately 7.5 years), 219 pairs of a child and his/her mother were reenrolled, and 172 pairs of them participated. In the follow-up study, which was conducted in May 2010, the participants were 172 child–mother pairs (at follow up, the children were approximately 7.5 years). In November 2011 (i.e., when the children were approximately 9 years), 117 of the 172 child–mother pairs were participated (response rate = 68%). The main reason for the high non-response rate was the participants’ change of address. We analyzed the data of 101 child–mother pairs as we had to exclude the data of the participants who had provided incomplete information. The mothers reported that their children did not have intellectual disability, epilepsy, and cerebral palsy. The characteristics of our sample are presented in Table 1.

**Table 1:** **The demographic data**.

	Mean	SD
Mother’s age (years)	41.16	4.10
Child’s age (years)	9.07	0.36
Brothers and sisters	2.26	0.88
Birth order	1.73	0.84
Sex (%)	Boy	girl
	53.47	46.53
Family structure (%)	Mother (and grandparents)	Mother and father
	0.99	83.17
	Parents and grandparent(s)	Others
	11.88	3.96

### Ethics Statement

The study protocol was approved by the Ethics Committees of the National Center of Neurology and Psychiatry (Protocol 26-144) and the National Center for Child Health and Development (Approval Number 530). The research was conducted in accordance with the Declaration of Helsinki. Written informed consent was obtained from each mother upon providing them with a detailed explanation of the study.

### Questionnaires

#### Parenting Behavior

The parenting behavior of the children’s mothers was assessed using the PS, and this was done when the children were 9 years (21). The Japanese version of the PS consists of two factors: laxness (8 items) and overreactivity (10 items) (27). The mothers self-rated each item using a 7-point Likert scale. The sum score of each factor was used for the analysis.

#### Strength and Difficulties Questionnaire

The mothers assessed their children’s behavior, using the Japanese version of the Strength and Difficulties Questionnaire (SDQ) (34, 35) when their children were 7.5 and 9 years. The SDQ consists of five subscales, each comprising five items: hyperactivity–­inattention, emotional symptoms, peer problems, conduct problems, and prosocial behavior (36). SDQ was also confirmed the same factor structure, good internal consistency, and test–retest reliability in a large Japanese sample (35). The first four subscales deal with children’s problematic behavior (i.e., difficulties), whereas the fifth subscale assesses prosocial behavior, which is positive behavior or a strength. The total difficulties score is calculated as the sum of the scores on the four subscales evaluating children’s problematic behavior. The children’s behavior was evaluated using a three-point Likert scale [0 (not true) to 2 (absolutely true)]. For the analysis, we used the total difficulties score and the sum score of prosocial behavior.

### Analysis

We conducted the statistical analyses, using R version 3.01 (37) for the preliminary analysis and AMOS ver. 19 (IBM Inc.) for the SEM. Table 2 shows the means, SDs, skewnesses, and kurtoses of all the variables. The skewnesses and kurtoses of the variables were in the range representing close to normal distribution (skewness, <2; kurtosis, <7) (38). Thus, we used parametric methods for the following analysis. To examine the relationship between each of the measures, we used Pearson product-moment correlation coefficients. We conducted SEM with maximum likelihood estimation to test our hypotheses. We then evaluated the model fit using the chi-squared test, the goodness-of-fit index (GFI), the adjusted goodness-of-fit index (AGFI), and the root mean square error of approximation (RMSEA). We considered the fit of the model to be acceptable when the chi-squared test showed non-significance, the GFI and AGFI were greater than 0.95, and the RMSEA was <0.05 (39). We used the Akaike information criterion (AIC) to compare the models. When we derived the final model based on these indices, we analyzed each path of the model using Wald tests.

**Table 2:** **Basic statistics**.

			Mean	SD	Skewness	Kurtosis
Age 7.5	Children	Prosocial behavior	7.13	2.30	−0.68	0.26
		Problematic behavior	9.45	4.93	0.95	0.54
Age 9	Children	Prosocial behavior	6.84	2.18	−0.40	−0.30
		Problematic behavior	8.86	5.13	1.06	0.66
	Mothers	Laxness	19.86	5.38	0.03	−0.63
		Overreactivity	33.55	8.77	0.28	−0.18

## Results

### Correlational Analysis

Table 3 shows the correlations between each of the variables. The children’s behavior (problematic and prosocial behavior) at 7.5 years was significantly correlated with their behavior at 9 years. The children’s problematic behavior at 7.5 years was significantly correlated with their mothers’ overreactivity at 9 years, whereas the children’s prosocial behavior was significantly correlated with their mothers’ laxness at 9 years. The mothers’ overreactivity was significantly correlated with their children’s problematic as well as prosocial behavior at 9 years. However, the correlations between the mothers’ laxness and their children’s behavior were not significant when the children were 9 years.

**Table 3:** **Correlational results**.

			Age 7.5		Age 9		
			Prosocial	Problem	Prosocial	Difficult	Overreact
Age 7.5	Children	Problem	−0.29**				
Age 9	Children	Prosocial	0.74***	−0.18			
		Problem	−0.22*	0.71***	−0.19		
	Mothers	Laxness	−0.21*	0.09	−0.14	0.11	
		Overreact	−0.20	0.27**	−0.28**	0.33**	0.13

*Prosocial, prosocial behavior; problem, problematic behavior; overreact, overreactivity*.

*p < 0.05, **p < 0.01, ***p < 0.001.

### Structural Equation Modeling

**Figure 2:**
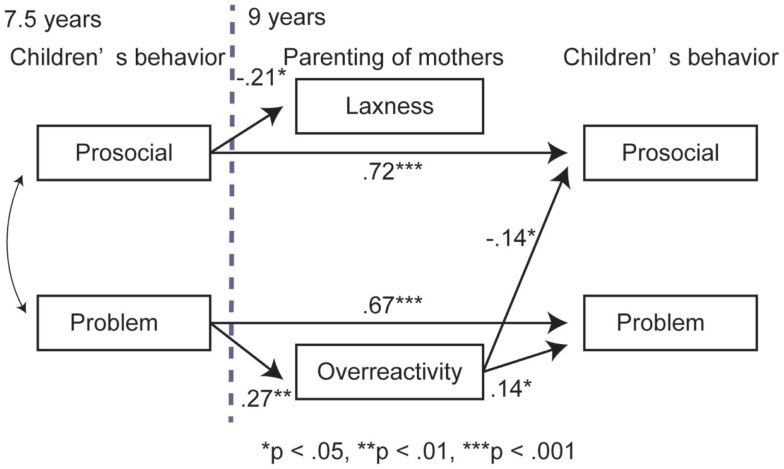
**Final model of the association between children’s behavior and mothers’ parenting**.

The original hypothetical model (Figure 1) was evaluated using SEM. Although the GFI and RMSEA showed an acceptable fit of the model, the AGFI fell below the acceptable level (GFI = 0.998, AGFI = 0.949, RMSEA = 0.000, AIC = 40.737). Hence, we modified the first model by removing non-significant paths and reanalyzed the model (Figure 2). All the indices exceeded the acceptable level and the AIC decreased (GFI = 0.985, AGFI = 961, RMSEA = 0.000, AIC = 30.572).

In the revised model (Figure 2), Wald tests revealed that all the paths were significant (*p* < 0.05). The children’s prosocial and problematic behavior at 7.5 years predicted their prosocial and problematic behavior, respectively, at 9 years. The children’s prosocial behavior at 7.5 years negatively predicted their mothers’ laxness at 9 years, whereas the children’s problematic behavior at 7.5 years positively predicted their mothers’ overreactivity at 9 years. The mothers’ overreactivity positively predicted their children’s problematic behavior at 9 years and negatively predicted their children’s prosocial behavior at 9 years.

## Discussion

We confirmed that children’s behavior (i.e., problematic and prosocial behavior) at 7.5 years predicted their behavior at 9 years. However, at 7.5 years, children’s problematic behavior and prosocial behavior were correlated with each other, whereas at age 9, there was no evidence of this correlation. These results implied that the mothers evaluated prosocial behavior and problematic behavior as dimensions that are closely related to each other when their children were 7.5 years, whereas when their children were 9 years, the mothers evaluated these two types of behaviors as independent behaviors. Children acquire an ability to think in light of the reciprocal interaction between their own and others’ perspectives at approximately 8–10 years (40), which indicates that prosocial behavior is more developed at 9 years than at 7.5 years. This later development of prosocial behavior might cause children’s prosocial and problematic behavior to be evaluated independently at a later age (i.e., 9 years).

We found that children’s problematic behavior at 7.5 years positively predicted the overreactivity of their mothers at 9 years. Previous study showed that the children’s problematic behavior increased the authoritarian and harsh parenting behavior of caregivers (14, 15), suggesting that severe behavioral problems in children triggered overreactive parenting in caregivers. Moreover, it is suggested that children’s characteristics strongly influenced the parenting style of their caregivers in Japan compared with that in other countries (20, 41). Hence, we considered that the influence of problematic behavior on the overreactivity was also found in this Japanese sample.

In addition, mothers’ laxness was positively predicted from their children’s prosocial behavior at 7.5 years. In a previous study, prosocial behavior was negatively associated with the maternal stress (42), suggesting that prosocial behavior may facilitate the interaction between children and their caregivers. We posit that this stimulates good interactions, thereby increasing the opportunity for the mother to instruct their children, which was associated with reduction of laxness.

In this study, the mothers’ overreactivity influenced both problematic and prosocial behavior in their children. Overreactive parenting has been reported to exacerbate children’s emotional regulation in Chinese population (8) as well as behavioral problems of ADHD children (5). Previous studies have also reported that harsh and assertive parenting reduced children’s prosocial behavior in South America (43). Mental health problem of Japanese adults was predicted by the authoritarian parenting style in childhood (30). Therefore, we considered overreactivity to be a dysfunctional parenting style in Japan.

On the other hand, we could not find any effects of the mothers’ laxness on their children’s behavior. In Japanese culture, ideally children become aware of their problematic behavior and modify it “without it being pointed out” (Iwanakutemo Wakaru in Japanese) by the caregiver or teacher. From this cultural background, Japanese mothers tended to avoid verbal instruction (18, 44). Thus, laxness may be partially accepted in Japan, although laxness is considered inappropriate in Western countries (21). Previous studies have shown that appropriate parenting styles are different among cultures, which mediates the influence of parenting behavior on children’s behavior (16, 17). Therefore, we suggested that the acceptance of laxness in Japan reduced the influence of laxness on children’s behavior in the present study, which is a unique characteristic of the parenting style of Japanese mothers.

In addition, we proposed the age of our sample concerning the influence of laxness. In previous studies, an inconsistent and permissive parenting style was associated with the development of psychological disorders (28) and violence (29) in adolescents. However, the participants of our sample were younger than those of the previous studies’ sample, and this could have been the reason for the current findings. Additional studies involving the analysis of longitudinal data from childhood to adolescence are required to examine the effect of parenting on children’s behavior in Japan.

In the previous study, we reported that a rich childbirth experience was associated with a decrease in parenting stress and anxiety (45), an increase in warmth of parenting, and an improvement of children’s behavior (32). Thus, children’s behavior and the parenting behavior may be explained using the childbirth experiences or/and the relationship between mothers and children from birth. However, our data were insufficient for constructing a highly detailed and longitudinal model, and the model was too complex to test. We proposed that clinicians need to consider the longitudinal relationship between mother and children from birth, and expected that the longitudinal model from birth will be confirmed in future.

There were limitations with regard to our conclusion. The parenting behavior and children’s behavior were evaluated by mothers in this study, meaning that the evaluations were influenced by the mother’s mental state. Furthermore, it was possible that mothers avoided evaluating their parenting behavior as inappropriate behavior. We assume that future studies will examine the association between caregivers and children in Japanese population using other indices, e.g., teacher’s rating questionnaire about children’s behavior, and the evaluation of the interaction between children and caregivers in the experimental setting. In addition, parenting behavior and children’s behavior were related to several factors, such as parenting behavior of other caregivers (8), socioeconomic status (46), and intellectual ability (47). More comprehensive models should be confirmed in future studies.

In our study, we confirmed the association between children’s behavior and the parenting of caregivers in Japanese population. The results showed that severe behavioral problems in children trigger inappropriate parenting in caregivers, which in turn, further exacerbates children’s behavior. Furthermore, there was no effect of laxness on children’s behavior, which may be associated with Japanese parenting style. These findings elucidate the association between children’s behavior and caregivers’ parenting in Japan. Previous studies intimate that there is a higher possibility of children and caregivers getting trapped in this vicious spiral when the children have developmental disorders (10–13). Thus, our study also highlights the importance of the implementation of clinical interventions to improve the relationship between children and caregivers.

## Author Contributions

All authors designed the work, wrote the manuscript, and approved the final version of the manuscript. KS, MK, KT, CM, and MI contribute for the data acquisition. KS and YK analyzed and interpreted the data.

## Conflict of Interest Statement

The authors declare that the research was conducted in the absence of any commercial or financial relationships that could be construed as a potential conflict of interest.

## References

[1] Turkheimer E Waldron M. Nonshared environment: a theoretical, methodological, and quantitative review. Psychol Bull (2000) 126(1):78–108. 10.1037/0033-2909.126.1.78 10668351

[2] Chronis AM Chacko A Fabiano GA Wymbs BT Pelham WE Jr. Enhancements to the behavioral parent training paradigm for families of children with ADHD: review and future directions. Clin Child Fam Psychol Rev (2004) 7(1):1–27. 10.1023/B:CCFP.0000020190.60808.a4 15119686

[3] McConachie H Randle V Hammal D Le Couteur A. A controlled trial of a training course for parents of children with suspected autism spectrum disorder. J Pediatr (2005) 147(3):335–40. 10.1016/j.jpeds.2005.03.056 16182672

[4] de Graaf I Speetjens P Smit F de Wolff M Tavecchio L. Effectiveness of the triple P positive parenting program on behavioral problems in children: a meta-analysis. Behav Modif (2008) 32(5):714–35. 10.1177/0145445508317134 18475003

[5] Baker BL Neece CL Fenning RM Crnic KA Blacher J. Mental disorders in five-year-old children with or without developmental delay: focus on ADHD. J Clin Child Adolesc Psychol (2010) 39(4):492–505. 10.1080/15374416.2010.486321 20589561 PMC2920761

[6] Smith LE Greenberg JS Seltzer MM Hong J. Symptoms and behavior problems of adolescents and adults with autism: effects of mother-child relationship quality, warmth, and praise. Am J Ment Retard (2008) 113(5):387–402. 10.1352/2008.113:387-402 18702558 PMC2826841

[7] Tully LA Arseneault L Caspi A Moffitt TE Morgan J. Does maternal warmth moderate the effects of birth weight on twins’ attention-deficit/hyperactivity disorder (ADHD) symptoms and low IQ? J Consult Clin Psychol (2004) 72(2):218–26. 10.1037/0022-006X.72.2.218 15065956

[8] Chang L Schwartz D Dodge KA McBride-Chang C. Harsh parenting in relation to child emotion regulation and aggression. J Fam Psychol (2003) 17(4):598–606. 10.1037/0893-3200.17.4.598 14640808 PMC2754179

[9] Singer GH. Meta-analysis of comparative studies of depression in mothers of children with and without developmental disabilities. Am J Ment Retard (2006) 111(3):155–69. 10.1352/0895-8017(2006)111[155:MOCSOD]2.0.CO;2 16597183

[10] Bebko JM Konstantareas MM Springer J. Parent and professional evaluations of family stress associated with characteristics of autism. J Autism Dev Disord (1987) 17(4):565–76. 10.1007/BF01486971 3680156

[11] Harvey E Danforth JS Ulaszek WR Eberhardt TL. Validity of the parenting scale for parents of children with attention-deficit/hyperactivity disorder. Behav Res Ther (2001) 39(6):731–43. 10.1016/S0005-7967(00)00052-8 11400716

[12] Mash EJ Johnston C. A comparison of the mother-child interactions of younger and older hyperactive and normal children. Child Dev (1982) 53(5):1371–81. 10.2307/1129028 7140436

[13] Braungart-Rieker J Garwood MM Stifter CA Compliance and noncompliance: the roles of maternal control and child temperament. J Appl Dev Psychol (1997) 18(3):411–28. 10.1016/S0193-3973(97)80008-1

[14] Laukkanen J Ojansuu U Tolvanen A Alatupa S Aunola K Child’s difficult temperament and mothers’ parenting styles. J Child Fam Stud (2014) 23(2):312–23. 10.1007/s10826-013-9747-9

[15] Scaramella LV Leve LD. Clarifying parent-child reciprocities during early childhood: the early childhood coercion model. Clin Child Fam Psychol Rev (2004) 7(2):89–107. 10.1023/B:CCFP.0000030287.13160.a3 15255174

[16] Lansford JE Chang L Dodge KA Malone PS Oburu P Palmerus K Physical discipline and children’s adjustment: cultural normativeness as a moderator. Child Dev (2005) 76(6):1234–46. 10.1111/j.1467-8624.2005.00847.x 16274437 PMC2766084

[17] Deater-Deckard K Dodge KA Bates JE Pettit GS Physical discipline among African American and European American mothers: links to children’s externalizing behaviors. Dev Psychol (1996) 32(6):1065 10.1037/0012-1649.32.6.1065

[18] Rothbaum F Pott M Azuma H Miyake K Weisz J. The development of close relationships in Japan and the United States: paths of symbiotic harmony and generative tension. Child Dev (2000) 71(5):1121–42. 10.1111/1467-8624.00214 11108082

[19] Azuma H Nihonjin no shitsuke to kyouiku: Hattatsu no nichibei hikaku ni motozuite [Japanese Discipline and Education: Comparison of Human Development in Japan and the United States]. Tokyo: Tokyo Daigaku Shuppankai (1994). In Japanese.

[20] Shikishima C Hiraishi K Yamagata S Neiderhiser JM Ando J Culture moderates the genetic and environmental etiologies of parenting a cultural behavior genetic approach. Soc Psychol Personal Sci (2013) 4(4):434–44. 10.1177/1948550612460058

[21] Arnold DS O’Leary SG Wolff LS Acker MM The parenting scale: a measure of dysfunctional parenting in discipline situations. Psychol Assess (1993) 5(2):137–44. 10.1037/1040-3590.5.2.137

[22] Matsumoto Y Sofronoff K Sanders MR. Investigation of the effectiveness and social validity of the triple P positive parenting program in Japanese society. J Fam Psychol (2010) 24(1):87–91. 10.1037/a0018181 20175613

[23] Gross D Fogg L Webster-Stratton C Garvey C Julion W Grady J. Parent training of toddlers in day care in low-income urban communities. J Consult Clin Psychol (2003) 71(2):261–78. 10.1037/0022-006X.71.2.261 12699021

[24] Leung C Sanders MR Leung S Mak R Lau J. An outcome evaluation of the implementation of the triple P-positive parenting program in Hong Kong. Fam Process (2003) 42(4):531–44. 10.1111/j.1545-5300.2003.00531.x 14979223

[25] Irvine AB Biglan A Smolkowski K Ary DV. The value of the parenting scale for measuring the discipline practices of parents of middle school children. Behav Res Ther (1999) 37(2):127–42. 10.1016/S0005-7967(98)00114-4 9990744

[26] Reitman D Currier RO Hupp SD Rhode PC Murphy MA O’Callaghan PM. Psychometric characteristics of the parenting scale in a head start population. J Clin Child Psychol (2001) 30(4):514–24. 10.1207/S15374424JCCP3004_08 11708239

[27] Itani T The Japanese version of the parenting scale: factor structure and psychometric properties. Jpn J Psychol (2010) 81(5):446–52. 10.4992/jjpsy.81.446 21226282

[28] Dwairy M Parental inconsistency: a third cross-cultural research on parenting and psychological adjustment of children. J Child Fam Stud (2010) 19(1):23–9. 10.1007/s10826-009-9334-2

[29] Miller JM DiIorio C Dudley W. Parenting style and adolescent’s reaction to conflict: is there a relationship? J Adolesc Health (2002) 31(6):463–8. 10.1016/S1054-139X(02)00452-4 12457579

[30] Uji M Sakamoto A Adachi K Kitamura T The impact of authoritative, authoritarian, and permissive parenting styles on children’s later mental health in Japan: focusing on parent and child gender. J Child Fam Stud (2014) 23(2):293–302. 10.1007/s10826-013-9740-3

[31] Dishion TJ French DC Patterson GR The development and ecology of antisocial behavior. In: Cicchetti D Cohen DJ, editors. Developmental Psychopathology, Vol. 2: Risk, Disorder, and Adaptation. Wiley Series on Personality Processes. Oxford: John Wiley & Sons (1995). p. 421–71.

[32] Suzuki K Kita Y Inoue Y Kaga M Misago C Takehara K Effects of a rich emotionally-satisfying childbirth experience of mothers on their later parental attitudes and behavior in school-age children. No To Hattatsu (2012) 44(5):368–73. 10.11251/ojjscn.44.368 23012865

[33] Takehara K Noguchi M Shimane T Misago C The development and evaluation of a childbirth experience scale (CBE-scale). Minzoku Eisei (2007) 73(6):211–24. 10.3861/jshhe.73.211

[34] Matsuishi T Nagano M Araki Y Tanaka Y Iwasaki M Yamashita Y Scale properties of the Japanese version of the strengths and difficulties questionnaire (SDQ): a study of infant and school children in community samples. Brain Dev (2008) 30(6):410–5. 10.1016/j.braindev.2007.12.003 18226867

[35] Moriwaki A Kamio Y. Normative data and psychometric properties of the strengths and difficulties questionnaire among Japanese school-aged children. Child Adolesc Psychiatry Ment Health (2014) 8(1):1. 10.1186/1753-2000-8-1 24444351 PMC3903008

[36] Goodman R Ford T Simmons H Gatward R Meltzer H. Using the strengths and difficulties questionnaire (SDQ) to screen for child psychiatric disorders in a community sample. Br J Psychiatry (2000) 177:534–9. 10.1192/bjp.177.6.534 11102329

[37] Development Core Team R R: A Language and Environment for Statistical Computing (2012). Available from: http://www.R-project.org/

[38] Bandalos DL Finney SJ Exploratory and confirmatory. In: Hancock GR Muller RO, editors. The Reviewer’s Guide to Quantitative Methods in the Social Sciences. New York; London: Taylor and Francis (2010). p. 93–114.

[39] Schermelleh-Engel K Moosbrugger H Müller H Evaluating the fit of structural equation models: tests of significance and descriptive goodness-of-fit measures. Methods Psychol Res (2003) 8:23–74.

[40] Selman RL The relation of role taking to the development of moral judgment in children. Child Dev (1971) 42(1):79–91. 10.2307/1127066 5549516

[41] Bornstein MH Haynes OM Azuma H Galperin C Maital S Ogino M A cross-national study of self-evaluations and attributions in parenting: Argentina, Belgium, France, Israel, Italy, Japan, and the United States. Dev Psychol (1998) 34(4):662–76. 10.1037/0012-1649.34.4.662 9681258

[42] Beck A Hastings RP Daley D Stevenson J Pro-social behaviour and behaviour problems independently predict maternal stress. J Intellect Dev Disabil (2004) 29(4):339–49. 10.1080/13668250400014509

[43] Hart CH DeWolf DM Wozniak P Burts DC. Maternal and paternal disciplinary styles: relations with preschoolers’ playground behavioral orientations and peer status. Child Dev (1992) 63(4):879–92. 10.2307/1131240 1505246

[44] Kazama M Hirabayashi H Karasawa M Tardif T Olson S Ambiguous parenting and four-year-olds’ understanding of others: a comparison between mothers in Japan and the US. Jpn J Dev Psychol (2013) 24(2):126–38.

[45] Takehara K Noguchi M Shimane T Misago C The positive psychological impact of rich childbirth experiences on child-rearing. Jpn J Public Health (2009) 56(5):312–21. 10.11236/jph.56.5_312 19588858

[46] Reiss F. Socioeconomic inequalities and mental health problems in children and adolescents: a systematic review. Soc Sci Med (2013) 90:24–31. 10.1016/j.socscimed.2013.04.026 23746605

[47] Olsson MB Hwang CP. Depression in mothers and fathers of children with intellectual disability. J Intellect Disabil Res (2001) 45(Pt 6):535–43. 10.1046/j.1365-2788.2001.00372.x 11737541

